# Exposure to tobacco imagery in popular films and the risk of ever smoking among children in southern India

**DOI:** 10.1136/tobaccocontrol-2019-055353

**Published:** 2020-09-08

**Authors:** Muralidhar M Kulkarni, Veena Ganesh Kamath, Asha Kamath, Sarah Lewis, Ilze Bogdanovica, Manpreet Bains, Jo Cranwell, Andrew Fogarty, Monika Arora, Gaurang P Nazar, Kirthinath Ballal, Rohith Bhagwath, John Britton

**Affiliations:** 1 Community Medicine, Kasturba Medical College, Manipal Academy of Higher Education, Udupi, India; 2 Department of Data Science, Prasanna School of Public Health, Manipal Academy of Higher Education, Udupi, India; 3 Division of Epidemiology and Public Health, UK Centre for Tobacco and Alcohol Studies, University of Nottingham, Nottingham, UK; 4 Department of Health, University of Bath, Bath, Somerset, UK; 5 Health Related Information Dissemination Amongst Youth (HRIDAY), New Delhi, India; 6 Health Promotion, Public Health Foundation of India, New Delhi, India

**Keywords:** advertising and promotion, low/middle income country, media, public policy, prevention

## Abstract

**Background:**

Exposure to smoking in films is a recognised cause of smoking uptake among children. In India, in an attempt to protect children, films containing smoking are required to include tobacco control messaging including audiovisual disclaimers, on-screen health warnings when tobacco imagery is displayed and antitobacco ‘health spots’ before and during the film. We report a study of the association between ever smoking and exposure to tobacco imagery in locally popular films among children in Udupi district of Karnataka state in southern India.

**Methods:**

A cross-sectional questionnaire survey of all students in grades 6–8 in schools in the Udupi district ascertained smoking status and potential confounders of smoking uptake, and whether children had seen any of 27 locally popular films we had coded and found to contain imagery of actual or implied tobacco use. Ever-smoking status was defined as any reported smoking of cigarettes, beedis or other tobacco products currently or at any time in the past. Independent effects on ever-smoking status were estimated using multiple logistic regression.

**Results:**

Of 46 706 students enrolled in grades 6–8 in 914 participating schools, 39 282 (84.1%) provided questionnaire responses sufficiently complete for analysis. Ever smoking was reported by 914 (2.3%) participants and in a mutually adjusted model was significantly related to age, male sex, living in a home where smoking is allowed, having parents or siblings who smoke, low paternal education, low levels of family wealth, low self-esteem, rebelliousness and poor school performance. After allowing for these effects, the odds of ever smoking were not increased among students who had seen any of the listed films containing tobacco imagery when included in the analysis as a binary exposure (OR 0.9, 95% CI 0.4 to 2.0), and decreased in relation to level of exposure graded into tertiles of tobacco intervals seen.

**Conclusions:**

In this cross-sectional study, children in southern India who had seen films containing tobacco imagery are no more likely to smoke than those who had not, indicating that the tobacco control messaging mandated by Indian law may be attenuating the effect of tobacco imagery in films on smoking uptake.

## Introduction

Smoking causes an estimated 7 million deaths each year,^1 2^ and around 80% of these deaths from tobacco now occur in low and middle-income countries.^1 2^ Smoking prevention is thus a global health priority, and the growing implementation of Framework Convention for Tobacco Control (FCTC) represents substantial progress in this respect.^2^ One of the key components of comprehensive tobacco control policy is protecting children from exposure to imagery that promotes smoking. While Article 13 of the FCTC prohibits paid-for tobacco advertising and other forms of promotion,^3^ it does not prevent unpaid inclusion of smoking and other tobacco imagery in films, television, on-demand video services and other media popular with children.

There is now growing recognition that watching films containing tobacco imagery causes incident smoking among children,^4–7^ but most of the available evidence for this effect arises from studies carried out in high-income countries.^8^ India, a lower middle-income country that is home to one in six of the global population, has a thriving film industry, and Indian films produced in the late 20th century were shown to contain high levels of tobacco imagery.^9^ Evidence on the effect of film smoking exposure on smoking among children in India is limited to a study of exposure to Bollywood films among secondary school-age children in Delhi^10^ in 2009. Since then the Indian government has introduced tobacco control legislation requiring that screenings of films containing tobacco imagery include an audiovisual (AV) disclaimer at the start of the film, health warnings during scenes containing tobacco and antismoking ‘health spots’ before and during the film.^11^ We now report a study of the association between smoking and exposure to tobacco imagery in locally popular films among children in a mixed urban and rural area of Karnataka state in southern India to determine whether smoking imagery in a range of Bollywood, international and local (southern Indian) films is associated with an increased risk of ever smoking.

## Methods

We used a cross-sectional questionnaire survey to measure smoking and exposure to smoking imagery in the media and other potential causal exposures and confounders in students in grades 6, 7 and 8 (aged between 10 and 15 years) attending any of the more than 700 government, 250 government-aided and 200 privately funded schools in the five educational administrative blocks (Udupi, Brahmavar, Karkala, Kundapura, Byndoor) in Udupi district of Karnataka state in India. We used a list comprising all government, private and aided schools obtained from the Udupi District Education Department to contact school principals and arrange a visit by a member of the survey staff to explain the study and obtain written consent for school participation. After obtaining the principal’s consent, researchers visited the school twice: first, to distribute a study information sheet for students and parental information and opt-out consent forms to all students in grades 6–8; and second, during a period of approximately 45 min scheduled into the schoolday 3–14 days later, to distribute questionnaires for completion by all consenting students whose parents did not exercise the opt-out. As school attendance rates are high we studied only those children present on the arranged study day; if for any reason (eg, heavy monsoon rains) fewer than 80% of students were in attendance, the survey was rescheduled. The student enrolment number issued by the education department was used as unique identifier for each participant. Data were collected between July 2017 and January 2018.

### Questionnaire design and study variables

The questionnaire elicited information on current and past use of cigarettes, beedis and a range of other smoked tobacco including cigars, cheroots, chillum, chutta and rolled cigarettes,^12^ with frequency of use (never; ever but not now; less than once a week; once a week; daily) using questions adapted from the Global Youth Tobacco Survey (GYTS),^13^ the UK Smoking, Drinking and Drug Use Survey,^14^ and Health Related Information Dissemination Amongst Youth’s Mobilizing Youth for Tobacco-Related Initiatives in India project.^15^ Questions on exposure to and awareness of tobacco products in retail outlets, including brand recognition, were drawn from the Nottingham Schools study.^16 17^ Awareness of health warnings and mass media campaigns were evaluated using questions from the GYTS India and Nottingham Schools surveys, adapted to include awareness of graphic and printed health warnings, and recall of exposure to tobacco control media campaigns.

Questions on exposure to tobacco imagery in films asked students whether they had seen any of 27 most popular films in Karnataka in the 2 years (2015 and 2016) preceding the study that we had previously interval coded and demonstrated to contain tobacco imagery.^11^ As previously reported,^11^ the most popular films were identified from national and local film distributor box-office ratings data, coded semiquantitatively using 5 min interval coding, and exposure quantified by summing the number of 5 min coded intervals containing tobacco imagery seen on the assumption of one complete viewing per film.^11^ Tobacco imagery was coded as actual use, implied use, tobacco paraphernalia and tobacco branding. The students were asked to mark if they had watched any of the listed films and as we had the details of number of tobacco intervals in each of the coded films, we could ascertain the exposure to tobacco imagery for each child. Film compliance with legal requirements under the Cigarettes and Other Tobacco Products Act (COTPA)^18 19^ regarding the inclusion of AV disclaimer, health spots of 30 s before and during the film and on-screen health warnings during scenes including smoking was also coded.^11^

Questions were included on the smoking policies adopted by the respondent’s school and in the family home, and on family smoking, peer smoking, self-esteem and rebelliousness.^20–22^ We measured socioeconomic status through a question on ownership of household goods, grouping participants into quintiles of family wealth.^23^ Other variables included age, gender, religion, academic grades in the past year, expectation of academic achievement, parents’ education and occupation. The questionnaire was piloted in a school in the neighbouring district and refined before use.

### Data analysis

Data were extracted from completed questionnaires into Microsoft Excel using Optical Mark Reader scanning and transferred into STATA V.9.2 software for analysis. Ever smoking was defined as any reported smoking of any tobacco product, currently or in the past. Associations between ever smoking and ordered or categorical variables were evaluated using logistic regression to estimate the effects of potential explanatory variables on the risk of smoking. Demographic variables were explored first and all that were significantly (p<0.05) associated with ever smoking were retained in the model. We then created a model which included all independently significant determinants of having ever smoked to then test the significance of measures of exposure to tobacco imagery in films first as a binary exposure (having seen or not seen a film containing at least one interval including tobacco imagery) and then as a graded variable in four categories (no exposure, and tertiles of those exposed). We also carried out an exploratory analysis to determine the effect on ever smoking of exposure to film smoking imagery in films according to the presence or absence of individual components of tobacco-free film rules required under COTPA. (Please refer to the online supplementary appendix for further details regarding study variables and analysis.)

10.1136/tobaccocontrol-2019-055353.supp1 Supplementary data

## Results

Of the total of 1214 schools in Udupi district listed by the education department we excluded 5 that had closed, 7 that were special schools for differently abled children and 281 lower primary and 2 high schools with no students in grades 6–8. We contacted the principals of the remaining 919 schools, and 914 (99.4%) of these consented to participate. Of the 46 706 students in grades 6–8 in 914 schools, 3066 were absent on the day of the survey, 271 declined to participate, 315 were excluded by parental opt-out and 6 students consented but did not complete a questionnaire. The five schools that declined to participate, and 507 of the 586 students who themselves or whose parents declined consent were all from private schools. The remaining 43 048 students (92.2%) completed the survey questionnaire. After excluding 3766 questionnaires with insufficient or otherwise unusable data, 39 282 questionnaires (representing 84.1% of the eligible students in consenting schools) were available for analysis. The 3766 questionnaires were either not completed by the students or had made contradicting responses and hence were not included for analysis. Respondents included similar proportions of males (51%) and females (49%), and of students from grades 6, 7 and 8 (32.5%, 33.6% and 33.9%, respectively). Most participants were of Hindu religion (83.3%) and from rural areas (80.1%).

Ever smoking was reported by 914 (2.3%) participants and in univariate analysis varied significantly by age, was more prevalent among male participants, those attending government-funded or part-funded schools, those who were not Hindus, who had family members or friends who smoked, lived in a home where smoking is allowed, attended a school where smoking was seen, whose parents were less educated, whose families were relatively poor and who were rebellious, reported low self-esteem and had poor performance at school (table 1). In a mutually adjusted model retaining independently significant associations, smoking was related to age, being male, living in a home where smoking is allowed, having parents, siblings or friends who smoke, low paternal education, low levels of family wealth, low self-esteem, rebelliousness and poor performance at school (table 1).

**Table 1:** Demographic and environmental associations with smoking in the study population, with univariate and independently significant mutually adjusted ORs

Characteristic	n (%)	Ever smokers (%)	Crude OR	P value	Adjusted OR*	P value
			(95% CI)		(95% CI)	
Age			<0.001			0.510
10	217 (0.6)	9 (4.1)	1		1	
11	5760 (14.7)	161 (2.8)	0.7 (0.3 to 1.3)		1.9 (0.6 to 6.1)	
12	12 932 (32.9)	328 (2.5)	0.6 (0.3 to 1.2)		1.7 (0.5 to 5.4)	
13	13 247 (33.7)	277 (2.1)	0.5 (0.3 to 1.0)		1.7 (0.5 to 5.4)	
14	6671 (17.0)	117 (1.8)	0.4 (0.2 to 0.8)		1.4 (0.5 to 4.6)	
15	455 (1.2)	22 (4.8)	1.2 (0.5 to 2.6)		2.0 (0.5 to 7.2)	
Gender				<0.001		0.003
Male	20 020 (51.0)	597 (3.0)	1.8 (1.6 to 2.1)		1.3 (1.1 to 1.6)	
Female	19 262 (49.0)	317 (1.6)	1		1	
School locality				0.773		–
Urban	7803 (19.9)	185 (2.4)	1.02 (0.9 to 1.2)			
Rural	31 479 (80.1)	729 (2.3)	1			
School type				<0.001		0.108
Government	16 786 (42.7)	416 (2.5)	1.4 (1.2 to 1.6)		1.2 (1.0 to 1.6)	
Aided	7584 (19.3)	227 (3.0)	1.7 (1.4 to 2.0)		1.3 (1.0 to 1.6)	
Private	14 912 (38.0)	271 (1.8)	1		1	
Religion				<0.001		0.068
Hindu	32 713 (83.3)	710 (2.2)	1		1	
Christian	2016 (5.1)	51 (2.5)	1.2 (0.9 to 1.6)		1.1 (0.8 to 1.6)	
Jain	152 (0.4)	7 (4.6)	2.2 (1.0 to 4.7)		1.7 (0.6 to 5.2)	
Muslim	4272 (10.9)	138 (3.2)	1.5 (1.3 to 1.8)		1.4 (1.1 to 1.8)	
Other	129 (0.3)	8 (6.2)	3.0 (1.5 to 6.1)		1.6 (0.6 to 4.2)	
Home smoking tobacco use allowed				<0.001		<0.001
No	35 400 (90.1)	588 (1.7)	1		1	
Yes	3882 (9.9)	326 (8.4)	5.4 (4.7 to 6.2)		2.8 (2.3 to 3.4)	
Family smoking tobacco use						
Father: Yes	4226 (10.8)	258 (6.1)	3.4 (2.9 to 3.9)	<0.001	1.9 (1.5 to 2.3)	<0.001
No	35 056 (89.2)	656 (1.9)	1		1	
Mother: Yes	386 (1.0)	99 (25.7)	16.1 (12.7 to 20.5)	<0.001	5.0 (3.4 to 7.2)	<0.001
No	38 896 (99.0)	815 (2.1)	1		1	
Siblings: Yes	721 (1.8)	127 (17.6)	10.3 (8.4 to 12.6)	<0.001	3.1 (2.2 to 4.2)	<0.001
No	38 561 (98.2)	787 (2.0)	1		1	
Others: Yes	6119 (15.6)	160 (2.6)	1.2 (1.0 to 1.4)	0.104		–
No	33 163 (84.4)	754 (2.3)	1			
Friends smoking tobacco use				<0.001		<0.001
None	34 430 (87.6)	352 (1.0)	1		1	
One	763 (1.9)	132 (17.3)	20.3 (16.3 to 25.1)		10.8 (8.1 to 14.4)	
Two	492 (1.3)	137 (27.9)	37.4 (29.9 to 46.7)		16.5 (12.2 to 22.4)	
Three	579 (1.5)	121 (20.9)	25.6 (20.4 to 32.0)		11.9 (8.7 to 16.2)	
Not sure	3018 (7.7)	172 (5.7)	5.9 (4.9 to 7.0)		4.0 (3.1 to 5.0)	
Smoking seen in school				<0.001		0.926
No	28 218 (71.8)	545 (1.9)	1		1	
Yes	11 064 (28.2)	369 (3.3)	1.8 (1.5 to 2.0)		1.0 (0.8 to 1.2)	
Mother’s education				<0.001		0.425
Illiterate	2457 (6.4)	94 (3.8)	4.3 (2.5 to 7.7)		1.1 (0.6 to 2.4)	
Primary	16 255 (42.0)	407 (2.5)	2.8 (1.6 to 4.8)		0.9 (0.5 to 1.8)	
High school	14 466 (37.4)	325 (2.2)	2.5 (1.5 to 4.3)		1.1 (0.6 to 2.1)	
Graduate	3964 (10.2)	53 (1.3)	1.4 (0.8 to 2.7)		1.0 (0.5 to 2.0)	
Postgraduate	1554 (4.0)	14 (0.9)	1		1	
Father’s education				<0.001		0.013
Illiterate	1757 (4.6)	79 (4.5)	7.0 (12.5 to 3.9)		2.8 (1.4 to 5.4)	
Primary	16 018 (41.5)	410 (2.6)	3.9 (6.7 to 2.3)		2.6 (1.4 to 4.9)	
High school	14 535 (37.7)	326 (2.2)	3.4 (5.9 to 2.0)		2.9 (1.6 to 5.4)	
Graduate	4129 (10.7)	67 (1.6)	2.5 (4.4 to 1.4)		2.5 (1.3 to 4.8)	
Postgraduate	2106 (5.5)	14 (0.7)	1		1	
Wealth quintile				<0.001		<0.001
Lower	7735 (19.7)	331 (4.3)	2.8 (2.3 to 3.4)		2.7 (2.1 to 3.6)	
Lower middle	7315 (18.6)	180 (2.5)	1.6 (1.3 to 2.0)		1.5 (1.1 to 2.0)	
Middle	8464 (21.6)	171 (2.0)	1.3 (1.0 to 1.6)		1.4 (1.0 to 1.9)	
Upper middle	7855 (20.0)	106 (1.3)	0.9 (0.7 to 1.1)		1.0 (0.7 to 1.4)	
Upper	7867 (20.1)	124 (1.6)	1		1	
Rebelliousness				<0.001		<0.001
No	24 500 (62.4)	311 (1.3)	1		1	
Mild	10 917 (27.8)	260 (2.4)	1.9 (1.6 to 2.2)		1.7 (1.4 to 2.1)	
Moderate	3396 (8.6)	267 (7.9)	6.6 (5.7 to 7.9)		3.8 (3.0 to 4.8)	
Severe	469 (1.2)	76 (16.2)	15.0 (11.5 to 19.7)		6.4 (4.4 to 9.5)	
High self-esteem				<0.001		0.001
Strongly agree	18 169 (46.3)	280 (1.54)	1		1	
Agree	8799 (22.4)	240 (2.7)	1.8 (1.5 to 2.1)		1.3 (1.0 to 1.6)	
Neither agree or disagree	6817 (17.4)	191 (2.8)	1.8 (1.5 to 2.2)		1.2 (1.0 to 1.6)	
Disagree	3099 (7.9)	123 (4.0)	2.6 (1.7 to 3.3)		1.8 (1.4 to 2.4)	
Strongly disagree	2398 (6.1)	80 (3.34)	2.2 (2.1 to 2.8)		1.4 (1.0 to 2.0)	
School performance				<0.001		0.001
Excellent	15 046 (38.3)	253 (1.7)	1		1	
Good	17 993 (45.8)	342 (1.9)	1.1 (0.9 to 1.3)		1.1 (0.9 to 1.3)	
Average	5337 (13.6)	249 (4.7)	2.9 (2.4 to 3.4)		1.4 (1.1 to 1.9)	
Below average	906 (2.3)	70 (7.7)	4.9 (3.7 to 6.4)		1.9 (1.3 to 2.8)	

*All p<0.05 after adjustment for gender, father’s education, family members smoking, friends smoking, wealth quintile, rebelliousness, self-esteem and school performance.

NS, not significant in mutually adjusted model.

### Exposure to smoking imagery or messaging and smoking behaviour

In an analysis in which single variables were added to the mutually adjusted model described in table 1, ever smokers were more likely than never smokers to report having participated in tobacco control activities, having seen or heard tobacco control messaging in the media or to have seen tobacco advertising (table 2). Almost all participants (38 698; 98.5%) reported having seen at least one of the 27 films containing smoking imagery listed in the questionnaire, and when added to the mutually adjusted model were not significantly more likely to be smokers than those who were unexposed, either before (unadjusted OR 2.0, 95% CI 0.9 to 4.2) or after (adjusted OR 0.9, 95% CI 0.4 to 2.0) adjustment for the above variables (data not shown). When exposure to tobacco imagery in films was included as a graded variable with exposure categorised into tertiles of the number of intervals containing tobacco imagery seen by the participant, there was no significant difference between tertiles but there was a significant negative trend in the odds of smoking across tertiles, which in the adjusted model declined from an OR of 1.2 (95% CI 0.5 to 2.6) in the first tertile of exposure to an OR of 0.7 (95% CI 0.3 to 1.6) in the highest tertile (table 2).

**Table 2:** Exposure to smoking imagery or messaging and smoking behaviour

Characteristic	n (%)	Ever smokers (%)	Model 1* Adjusted OR* (95% CI)	P value	Model 2† Adjusted OR (95% CI)	P value
Class on health hazards of tobacco				0.003		
Yes	7917 (20.2)	243 (3.1)	1			
No	17 284 (44.0)	363 (2.1)	0.8 (0.6 to 0.9)			
Not sure	14 081 (35.8)	308 (2.2)	0.7 (0.6 to 0.9)			
Participated in antitobacco activity				<0.001		<0.001
Yes	7675 (19.5)	288 (3.8)	1		1	
No	31 607 (80.5)	626 (2.0)	0.6 (0.5 to 0.7)		0.6 (0.5 to 0.7)	
Heard or seen antitobacco media messages				<0.001‡		<0.001‡
None	14 106 (35.9)	291 (2.1)	1		1	
1–5	10 291 (26.2)	143 (1.4)	0.7 (0.5 to 0.9)		0.7 (0.5 to 0.8)	
6–10	3597 (9.2)	184 (5.1)	1.8 (1.4 to 2.4)		1.7 (1.4 to 2.2)	
>10	11 288 (28.7)	296 (2.6)	1.2 (1.0 to 1.5)		1.1 (0.9 to 1.4)	
Exposure to tobacco advertisements				0.442		
Yes	30 111 (76.7)	743 (2.5)	1.1 (0.9 to 1.3)			
No	9171 (23.3)	171 (1.9)	1			
Exposure to tobacco interval in movies				<0.001‡		<0.001‡
0	584 (1.5)	7 (1.2)	1		1	
1–48	12 079 (30.7)	363 (3.0)	1.2 (0.6 to 2.7)		1.2 (0.5 to 2.6)	
49–83	13 277 (33.8)	270 (2.0)	0.9 (0.4 to 1.9)		0.9 (0.4 to 1.9)	
>83	13 342 (34.0)	274 (2.1)	0.8 (0.3 to 1.7)		0.7 (0.3 to 1.6)	

*Model 1. All p<0.05 after adjustment for age, gender, father’s education, family members smoking, friends smoking, wealth quintile, rebelliousness, self-esteem and school performance.

†Model 2. Mutually adjusted with exclusion of non-significant (NS) variables in this table.

‡P value for trend.

In an exploratory analysis of exposure to tobacco imagery in films categorised in relation to their compliance with COTPA smoke-free film rules it was observed that exposure to an AV disclaimer at the start of the film was associated with a significantly lower risk of smoking, but there were no significant associations between smoking and other measures of compliance (table 3).

**Table 3:** Exposure to components of smoke-free film rules and ever smoking in the study population

Compliance to smoke-free film rules	n (%)	Ever smoker (%)	Crude OR (95% CI)	P value	Adjusted OR* (95% CI)	P value
AV disclaimer at the start of the film				0.039		0.022
No	7171 (18.5)	192 (2.7)	1.2 (1.0 to 1.4)		1.3 (1.0 to 1.5)	
Yes	31 527 (81.5)	715 (2.3)	1		1	
Health spot at the start of the film				0.861		0.969
No	2463 (6.4)	59 (2.4)	1.0 (0.8 to 1.3)		1.0 (0.7 to 1.4)	
Yes	36 235 (92.2)	848 (2.3)	1		1	
Health spot in the middle of the film				0.109		0.391
No	3234 (8.4)	89 (2.8)	1.2 (1.0 to 1.5)		1.1 (0.9 to 1.5)	
Yes	35 464 (91.6)	818 (2.3)	1		1	
Any static warning messages				0.329		0.346
No	223 (0.6)	3 (1.3)	0.6 (0.2 to 1.8)		0.5 (0.1 to 2.1)	
Yes	38 475 (99.4)	904 (2.3)	1		1	
COTPA-compliant static warning messages				0.415		0.261
No	7944 (20.5)	196 (2.5)	1.1 (0.9 to 1.3)		1.1 (0.9 to 1.4)	
Yes	30 754 (79.5)	711 (2.3)	1		1	

*All p<0.05 after adjustment for age, gender, father’s education, family members smoking, friends smoking, wealth quintile, rebelliousness, self-esteem and school performance.

AV, audiovisual; COTPA, Cigarettes and Other Tobacco Products Act.

## Discussion

This study demonstrates that ever smoking was uncommon among children in grades 6–8 in schools in Karnataka state in southern India, but more likely to have occurred among males and among children who live with smokers or have friends who smoke, live in low educational level and low-income families and in homes where smoking is allowed, who are more rebellious and have low self-esteem and poor school performance. After allowing for these effects, which are consistent with those previously reported in India^24^ and widely established in studies of smoking among young people elsewhere in the world,^6^ participants in this study were also independently more likely to have ever smoked if they had heard or seen tobacco control messages or participated in other antitobacco activities, but no more likely to smoke if they had seen any one or more of 27 locally popular films we had previously demonstrated to contain tobacco imagery.^11^ There was also evidence of a significant negative relation between smoking risk and level of exposure to smoking imagery in films.

The association with exposure to tobacco control activities and messaging is likely to reflect reverse causation, whereby young people who smoke are more likely to recall tobacco control messages, but our findings in relation to the effect of film imagery exposure on smoking risk conflict with existing evidence, predominantly from high-income countries, that exposure to smoking imagery in film is consistently associated with a greater likelihood of smoking.^8^ These associations can also be linked to use of outcome variable in our analysis, which is ever smoking and not current smoking as in most similar studies. Therefore, while our general findings indicate that children in this area of India take up smoking for similar reasons as those in richer countries,^6^ they also indicate that the effect of exposure to tobacco imagery in films in southern India may have less effect on smoking than in other countries. The most likely explanation for this is that the smoke-free film measures required in India since 2012 protect young people against harm from exposure to tobacco imagery.

India is a young nation, with half of the 1.3 billion population aged under 27 years.^25^ In common with many other low and middle-income countries, the prevalence of smoking in India is low in relation to that in high-income countries, and especially so among women.^26^ Furthermore, smoking prevalence has, over recent years, been falling. Despite this trend, however, rapid population growth is generating increasing numbers of smokers, particularly among younger age groups,^27 28^ presaging a major future epidemic of tobacco-related death and disability.^29 30^ If India is to avoid following the world’s high-income countries down a path of major damage to public health from tobacco use^31^ it is essential that the determinants of initiating tobacco use in India are understood. The estimate in the present study of prevalence of ever smoking among young people in grades 6–8 of education, most of whom were aged 11–14 years, is based on a large population sample comprising 85% of eligible children in schools in the study district. It is therefore highly representative. The prevalence of ever smoking, at 2.3%, in our survey conducted in 2017 was lower than the 4.4% national prevalence of current smoking reported in the 2009 GYTS in India,^32^ but the participants in our study were younger than GYTS participants, and from only one part of a country in which marked regional variations in prevalence have already been documented.^24^ Our figure is however broadly consistent with an earlier estimate of 4% ever use of smoked tobacco in the GYTS survey carried out in Karnataka in 2004^33^ and a study in similar age group students in Noida.^34^ Our figures do not include use of smokeless tobacco, which is in common use in India,^35^ and will be reported separately. The prevalence of current smoking in our sample was inevitably lower than that of ever smoking, and in view of the relatively small numbers involved we used ever smoking as our primary outcome to maximise study power.

Our finding that participants who had seen tobacco imagery in a range of locally popular films were not more likely to be ever smokers conflicts with a substantial literature demonstrating that exposure to smoking in films increases the risk of smoking,^8^ an association that is accepted by the US Surgeon General and other authorities to be causal.^4–7^ Our finding is therefore unexpected, suggesting that it is either a false negative or indicates that the effects of film exposure are getting diluted due to tobacco-free film and TV rules. The mediating role of these rules in explaining an association between tobacco use and tobacco imagery needs to be further investigated. If so, then the most likely explanation is attenuation by the COTPA tobacco-free film rules introduced in 2012,^11^ and our exploratory analysis suggests that of the several components of these measures, the presence of an AV disclaimer at the start of the film may be particularly important in this respect. However, the possibility that the effect of film tobacco imagery is offset by tobacco control messaging in films as a requirement of Indian tobacco control laws is consistent with the finding of an effect of film exposure on smoking among children in Delhi, conducted before the new tobacco control measures were introduced.^10^

One of the potential limitations of this study is that the models include variables which are correlated with each other, and while we have taken a strategic approach to deciding which variables to include in multivariate models, there remains a possibility of multicollinearity (ie, that effects may be dependent on the presence or absence of another correlated variable in the model). This study is, however, only a baseline description of findings in a cohort of children that has been followed prospectively over time, and we will report the prospective findings on the association between exposure to smoking imagery in films and subsequent smoking initiation in this cohort in further studies. We present these findings therefore as preliminary evidence that Indian tobacco control measures may have been successful in eradicating this important influence on smoking uptake.

**What this paper adds**

- Exposure to smoking imagery in films has been shown to increase the risk of smoking uptake among adolescents and is causal.
- This article is the first to explore the impact of Indian smoke-free laws on smoking uptake among adolescents in southern India.
- The study shows that children are no more likely to smoke if they have seen films containing smoking imagery.
- This paper also suggests that Indian smoke-free film rules might be successful in attenuating the impact of smoking imagery on children.

## Data Availability

Data are available upon reasonable request. All the data are stored in anonymised form with the Co-PI of the research project. The metadata could be shared after obtaining regulatory approvals based on the purpose of requirement.
