# Supplementary material for: Exposure to tobacco imagery in popular films and the risk of ever smoking among children in southern India
Source: Tob Control. 2020 Sep 8;30(5):560–6. doi: 10.1136/tobaccocontrol-2019-055353 (PMC8394755; doi:10.1136/tobaccocontrol-2019-055353)
Supplement: Supplementary data [file tobaccocontrol-2019-055353supp001.pdf]

Key study variables:

Dependent variable:

Ever tobacco use: The question on tobacco use frequency comprised of options as never; ever but not now; less than once a week; once a week and daily. Ever smoking was defined as any reported smoking of any tobacco product, currently or in the past. Thus any students marking any one of the last four options was considered as an ever-smoker.

Independent variables:

Tobacco imagery in films; Educational class on health hazards of tobacco; Participation in anti-tobacco activities; Heard or seen anti-tobacco messages on radio/TV, posters, newspapers / magazines; Observed tobacco advertisements on public transport vehicles, public places, internet, radio/TV, Newspapers/ Magazines.

Tobacco imagery in films: We coded 47 popular films that included films in Hindi, English, Kannada, Tulu, Tamil, Telugu and Malayalam using 5 minute interval coding method recording the presence or absence in each interval of:

Actual Use: Use of tobacco onscreen by any character

Implied Use: Implied but not actual use on screen, verbal or non-verbal.

Tobacco Paraphernalia: Presence of tobacco products or related materials such as matchboxes, lighters, ashtrays and smoking signage.

Brand Appearance: Presence of clear, unambiguous, tobacco branding.

Among the 47 films coded, 27 films had either actual or implied tobacco use which in our case was smoking scenes only and were included in the questionnaire. The students were asked to mark if they had watched any of the 27 films. As we had the details of number of tobacco intervals in each of the coded films we could ascertain the exposure to tobacco imagery for each child.

Components of smoke free film rules:

AV disclaimers: The AV disclaimers have to be present in the beginning and middle of the films and we noticed that only 5(18.5%) films had them in the beginning of the film while none had in the middle.

Health spots: These include a short film on tobacco hazards for 20 seconds and have to be present in the beginning and middle of the film and was present in 10(37.0%) and 5(18.5%) films respectively.

Static messages: This component includes warning messages placed prominently at the bottom of the screen, in black font on white background, and used the messages "Smoking causes cancer" or "Smoking kills" for smoked tobacco, or "Tobacco causes cancer" or "Tobacco kills" for chewing and other smokeless forms of tobacco in the language of the film. These messages were observed and categorized as present or absent and if present, whether it was as per rules.

If a child had watched a film with any component of smoke free film rules, such children were considered to be exposed to that specific measure like AV disclaimer or Health spots or Static messages and the association with ever smoking was analyzed.

Educational class on health hazards of tobacco: The student was asked whether he/she had a class on educational class on health hazards of tobacco in the past one year. The options included yes; no; and not sure.

Participation in anti-tobacco activities: We asked whether a student was involved in any anti-tobacco activity in the past one year.

Anti-tobacco messages: The questions were asked separately whether a student has heard or seen anti-tobacco messages on (a) radio/ TV (b) posters (c) Newspapers/ Magazines. The options included none, one to five messages, six to ten messages and more than ten messages. The response from these questions were combined to ascertain the exposure to anti-tobacco messages.

Tobacco advertisements: The students had to answer questions if they have noticed tobacco advertisements on public transport vehicles, public places, internet, radio/TV and Newspapers/ Magazines separately. The options were yes or no. The responses were then combined to assess whether a student was exposed to tobacco advertisement.

Control variables:

Age, gender, religion, school type, school locality, Fathers education, mothers education, Home smoking allowed, family members smoking, friends smoking, rebelliousness, self-esteem and school performance.

School type: Schools in Indian context are usually classified based on the source of funding and classified as Government, if the funding is by the state; Aided, when the funding is by the state but managed by a private agency; Private, if the school is self-funded without any funding by the state.

School locality: This was classified as urban or rural as per the Census 2011 guidelines.

Rebelliousness: We asked children a set of three questions based on a previous study conducted in SEAR countries. The questions included whether they ignore rules, - do things their parents wouldn't want them to do, - get into trouble with authorities at school work or other places. The students had to mark as never, sometimes or often for these questions that were scored as 0, 1, and 2 and an aggregate score generated to classify children as having No, Mild, moderate and severe rebelliousness.

Self-esteem: This was assessed by a single item questionnaire based on whether they think they have high self-esteem and were asked to respond on a five point Likert scale which included options from strongly agree to strongly disagree.

Age, gender, religion, Fathers education, Mothers education, family and friends smoking: These responses were elicited by providing relevant options and children had to mark the most appropriate option.
